# Antibiotic Susceptibilities of Bacteria Isolated within the Oral Flora of Florida Blacktip Sharks: Guidance for Empiric Antibiotic Therapy

**DOI:** 10.1371/journal.pone.0104577

**Published:** 2014-08-11

**Authors:** Nathan R. Unger, Erich Ritter, Robert Borrego, Jay Goodman, Olayemi O. Osiyemi

**Affiliations:** 1 Department of Pharmacy Practice, Nova Southeastern University College of Pharmacy, Palm Beach Gardens, Florida, United States of America; 2 Department of Mathematics and Statistics, University of West Florida, Pensacola, Florida, and Shark Research Institute, Princeton, New Jersey, United States of America; 3 Division of Trauma, St. Mary’s Medical Center, West Palm Beach, Florida, United States of America; 4 Division of Laboratory Services, St. Mary’s Medical Center, West Palm Beach, Florida, United States of America; 5 Triple O Medical Services, P.A., West Palm Beach, Florida, United States of America; Charité, Campus Benjamin Franklin, Germany

## Abstract

Sharks possess a variety of pathogenic bacteria in their oral cavity that may potentially be transferred into humans during a bite. The aim of the presented study focused on the identification of the bacteria present in the mouths of live blacktip sharks, *Carcharhinus limbatus*, and the extent that these bacteria possess multi-drug resistance. Swabs were taken from the oral cavity of nineteen live blacktip sharks, which were subsequently released. The average fork length was 146 cm (±11), suggesting the blacktip sharks were mature adults at least 8 years old. All swabs underwent standard microbiological work-up with identification of organisms and reporting of antibiotic susceptibilities using an automated microbiology system. The oral samples revealed an average of 2.72 (±1.4) bacterial isolates per shark. Gram-negative bacteria, making up 61% of all bacterial isolates, were significantly (p<0.001) more common than gram-positive bacteria (39%). The most common organisms were *Vibrio spp*. (28%), various coagulase-negative *Staphylococcus spp*. (16%), and *Pasteurella spp.* (12%). The overall resistance rate was 12% for all antibiotics tested with nearly 43% of bacteria resistant to at least one antibiotic. Multi-drug resistance was seen in 4% of bacteria. No association between shark gender or fork length with bacterial density or antibiotic resistance was observed. Antibiotics with the highest overall susceptibility rates included fluoroquinolones, 3^rd^ generation cephalosporins and sulfamethoxazole/trimethoprim. Recommended empiric antimicrobial therapy for adult blacktip shark bites should encompass either a fluoroquinolone or combination of a 3^rd^ generation cephalosporin plus doxycycline.

## Background

Florida consistently boasts the highest number of shark attacks in the world, accounting for nearly one-third of all incidents in 2013 with about 20% caused by blacktip sharks (*C. limbatus*) based on witness and victim accounts, and to some extent scientific determination [1]–[3]. Although these bites within Florida waters are rarely fatal, victims of severe bites are at risk for subsequent infection and related complications due to entry of bacteria from the shark’s oral cavity into the open wound. As such, timely administration of appropriate antibiotics and wound care is of the utmost importance [4]–[10]. In general, empiric antimicrobial therapy should provide adequate coverage against anticipated pathogens, accounting for drug resistance. In the case of a shark bite, antibiotic therapy should target the bacteria transferred from the oral flora of the shark into the victim’s wound. Current recommendations for the use of prophylactic antimicrobials for shark bites consist of either monotherapy with or some combination of a parenteral third-generation cephalosporin, trimethoprim–sulfamethoxazole, aminoglycosides, or ciprofloxacin [9]–[11]. Carbapenems may serve as the empiric treatment of choice for an established wound infection or evidence of sepsis [9]–[11]. However, with the global rise of antimicrobial resistance, these recommendations may no longer be appropriate.

Several studies have identified the presence of pathogenic bacteria (e.g., *Vibrio spp., Pseudomonas spp., Citrobacter spp.*) in marine animals, including sharks [12]–[17]. Of great interest is the degree of antibiotic resistance observed in these studies, which may be related to the pumping of sewage into the waters [12]–[15], [17]–[20]. In Brazil, oral samples collected post-mortem from bull sharks (*C. leucas*) and tiger sharks (*Galeocerdo cuvier*) revealed a high-level of antimicrobial resistance among *Enterobacteriaceae*, including 20% of *Proteus mirabilis* resistant to imipenem, a broad-spectrum antibiotic [15]. Additionally, samples from the cloaca-anus of nurse sharks (*Ginglymostoma cirratum*), bull sharks, blacktip sharks and lemon sharks (*Negaprion brevirostris*) also demonstrated resistance to several tested antibiotics, including approximately 20–35% resistance amongst amikacin, ceftazidime and piperacillin [12]. Due to their clinical importance in the treatment of serious multi-drug resistant gram-negative infections, the degree of resistance described in the aforementioned antibiotics is concerning.

Previous investigations into the bacteria content and antibiotic resistance within the anatomy of sharks either focused on oral samples of deceased sharks or the cloaca- anal swabs. However, no study to date has examined the oral flora of live sharks for the presence of antibiotic resistant bacteria. The primary objective of our study was to 1) identify the bacteria present in the oral flora of blacktip sharks, and 2) quantify the extent of antibiotic resistance in these live sharks in order to provide definitive microbiologically based guidance for appropriate empiric antimicrobial therapy for respective victims.

## Methods

### Ethics Statement

Research was conducted in accordance with a special activity license (License#SAL-12-1429C-SRP) issued by the state of Florida and with approval of the Institutional Animal Care and Use Committee (IACUC Control#042-398-13-0102) at Nova Southeastern University. All sharks caught for the purpose of the presented research were released unharmed and all efforts were made to minimize suffering of the sharks sampled; moreover, no shark was killed, harvested or sold as a consequence of this study.

### Shark capture and sampling

**Figure 1:**
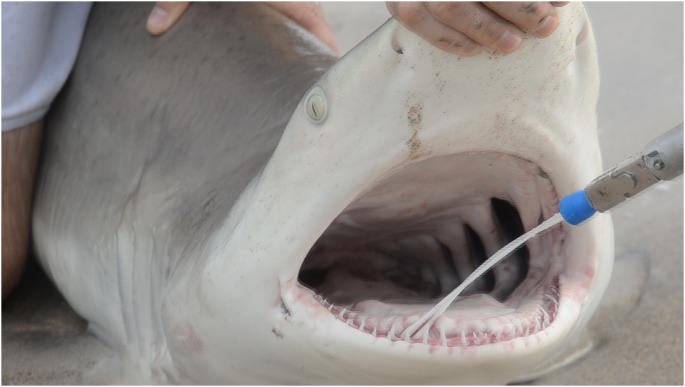
Swabbing the oral cavity of a blacktip shark.

Blacktip sharks were caught with a surf rod and a sixty-five pound fishing line with a 12/0 circle hook off of beaches in Martin and Palm Beach Counties in Florida, which have been previous identified by research as high-risk cluster areas of shark attacks along the Florida coast [21]. Captures took place between February and April 2013. Once landed, the mouth of the shark was opened, upper and lower teeth sampled, inside and outside, including the gums using a remote swabbing device (GlobePharma, Inc, North Brunswick, NJ) with a BBL CultureSwab Plus (Becton, Dickinson and Company, Sparks, MD) securely attached to the end (Figure 1). Identification of shark gender and measurement of fork length was performed. Fork length reflects the distance from the snout of the shark to the fork of the tail.

### Microbiological procedures

All swabs were placed in a cooler with ice packs and transported to the microbiology lab at St. Mary’s Medical Center within 48 hours. After receipt of each swab, samples were streaked on several different agar plates, including sheep blood, MacConkey, chocolate and CNA media. Aerobic and anaerobic media were incubated at 35°C for 24 hours and 48 hours, respectively. All microbiological work-up was conducted in accordance with the policies and procedures governed by the College of American Pathologists (CAP), the American Society for Microbiology (ASM) and the Clinical and Laboratory Standards Institute (CLSI). Organisms were identified and antibiotic susceptibilities were performed using an automated microbiology system, the Siemens Microscan Walk-Away 96 SI. Antibiotics used for susceptibility testing can be found in Table 1. If susceptibility testing could not be performed on the automated system (due to growth characteristics of the particular organism), the manual Kirby Bauer disk-diffusion was used and interpreted in accordance with CLSI M100 Performance Standards for Antimicrobial Susceptibility Testing. For the purpose of this study, bacteria lacking standardized antibiotic susceptibility interpretation (e.g., *Pasteurella sp., Bacillus sp., Micrococcus sp*. and alpha-hemolytic *Streptococcus sp*.), as outlined in the CLSI guideline, were considered routinely susceptible to typical recommended agents. Additionally, if antibiotic susceptibility testing indicated an organism was intermediate, it was deemed resistant. Multi-drug resistance was defined as resistance to three or more different antibiotic classes, excluding antibiotics or antibiotic classes that select bacteria possess intrinsic resistance against (e.g., *Enterobacter cloacae* is innately resistant to 1^st^ and 2^nd^ generation cephalosporins) [22].

**Table 1:** Antibiotics tested against isolated bacteria for susceptibility.

Gram-negative bacteria	Gram-positive bacteria	Gram-negative and positive bacteria
Aztreonam (AZT)	Clindamycin (CLIN)	Ampicillin (AMP)
Cefazolin (CFZ)	Daptomycin (DAP)	Ampicillin/sulbactam (A/S)
Cefotaxime (CFT)	Erythromycin (ERY)	Ceftriaxone (CFT)
Ceftazidime (CTZ)	Linezolid (LZD)	Ciprofloxacin (CIP)
Cefuroxime (CFM)	Oxacillin (OXA)	Sulfamethoxazole/trimethoprim (S/T)
Imipenem (IMI)	Penicillin (PCN)	
Piperacillin/tazobactam (P/T)	Rifampin (RIF)	
Tigecycline (TIGE)	Tetracycline (TET)	
Tobramycin (TOB)	Vancomycin (VAN)	

### Statistical analysis

Types of bacteria and antibiotic susceptibilities are reported as frequencies, and the number of bacteria per shark is expressed as a mean (± standard deviation). A one and two-sample t-test was applied to bacteria composition of all the samples and compare mean number of bacteria and resistant antibiotics per shark by gender, as appropriate. A Pearson’s Chi-square test was used to compare the proportion of antibiotics with resistance amongst gram-positive and gram-negative bacteria. The relationship between fork length of the shark with bacterial density and antibiotic resistance was tested using Pearson correlation coefficients. Additionally, antibiotic resistance, bacterial composition (i.e. gram-positive, gram-negative) and mean number of bacteria were extracted from the study by Interaminense et al. [15] and compared to data from this study using a two-sample t-test. A p-value of less than 0.05 was considered significant.

## Results

A total of 19 blacktip sharks were caught and swabbed; however, one sample was excluded due to contamination prior to transportation to the lab. Twelve sharks were male, four were female and two sharks did not have their gender documented. The fork length spectrum of the sharks ranged from 122 to 168 cm with an average of 146 cm (±10.54). Using published growth curves for blacktip sharks, the range and mean fork length suggest all sampled sharks were mature sharks at least 8 years of age [23]. A total of 49 bacteria species were isolated consisting of 22 different genera. Each shark yielded an average of 2.72 (±1.4, 95% CI 2.32–3.13) bacterial isolates, which is significantly less (p<0.001) than the mean number of bacteria found in tiger sharks (9±0) and bull sharks (9±0) in Brazil as reported by Interaminense et al. (Table 2) [15].

**Table 2:** Comparison bacteriology and antibiotic resistant within the oral flora of multiple shark species using data from previously published literature [15].

Study	Shark species (n)	Bacterialisolates, n	Bacterial isolatesper sample,mean±SD (95% CI)	p-value	Gram-positivebacteria, % (95% CI)	Gram-negativebacteria, % (95% CI)	p-value	Antibioticresistance^a^, % (95% CI)	p-value
**Current study**	Blacktip (18)	49	2.72±1.4 (2.32–3.13)	-	39 (25–53)	61 (47–75)	-	22 (17–27)	-
**Interaminense et al (2010)**	Tiger (5)	45	9±0 (N/A)	<0.001	27 (13–40)	73 (60–87)	0.216	22 (17–26)	0.882
**Interaminense et al (2010)**	Bull (4)	36	9±0 (N/A)	<0.001	17 (4–30)	83 (71–96)	0.027	17 (13–22)	0.162

a Calculated using susceptibility data for only the antibiotics tested in both current study and Interaminense et al. If antibiotic susceptibility testing indicated an organism was intermediate, it was deemed resistant.

Gram-negative bacteria comprised a significantly higher proportion of the isolated bacteria compared to gram-positive bacteria (61 vs 39%, p<0.001) (Table 3). Gram-negative bacteria predominately consisted of *Vibrio alginolyticus* (14%), other *Vibrio sp*. (14%) and *Pasteurella sp.* (12%). The most common gram-positive organisms included a composite of various coagulase-negative *Staphylococcus* spp. (16%) [i.e. *S. epidermidis, S. scuiri, S. cohnii-urea, S. hominis], S. aureus* (8%) and *Bacillus sp.* (6%). When bacterial composition was compared to the aforementioned Interaminense et al. study [15], there was no difference in the proportion of gram-positive and gram-negative bacteria between blacktip and tiger sharks (p = 0.216); however, a significant difference was seen regarding bull sharks (p = 0.027). *Enterobactericeae* comprised 17% (95% CI 3–31) of gram-negative organisms isolated from blacktip sharks, which was significantly less than bull sharks (97%, 95% CI 90–100, p<0.001) and tiger sharks (79%, 95% CI 64–94, p<0.001) [15]. No anaerobic bacteria were found in the oral samples of the captured blacktip sharks. When stratified by gender, no difference was seen between females and males in bacterial composition (Table 4). Additionally, fork length was not associated with mean number of bacteria (r = 0.222, p = 0.382), gram-positive bacteria (r = 0.221, p = 0.384) or gram-negative bacteria (r = 0.058, p = 0.822).

**Table 3:** Bacteria isolated in the oral cavity of blacktip sharks^a^.

Gram-negative bacteria	n (%)	Gram-positive bacteria	n (%)
*V. alginolyticus*	7 (14)	*Staphylococcus aureus*	4 (8)
*Vibrio sp.*	7 (14)	*Bacillus sp.*	3 (6)
*Pasteurella sp.*	6 (12)	Coagulase-negative *Staphylococcus sp.*	3 (6)
*Pasteurella aerogenes*	2 (4)	*S. epidermidis*	2 (4)
*Enterobacter cloacae*	2 (4)	*S. scuiri*	1 (2)
*Enterobacter aerogenes*	1 (2)	*S. hominis*	1 (2)
*Escherichia coli*	1 (2)	*S. cohnii-urea*	1 (2)
*Klebsiella sp.*	1 (2)	*Streptococcus bovis*	1 (2)
*Moraxella sp.*	1 (2)	*Micrococcus sp.*	1 (2)
*Shewanella putrefacians*	1 (2)	*Enterococcus faecium*	1 (2)
*Pseudomonas sp.*	1 (2)	alpha-hemolytic *Streptococcus sp.*	1 (2)
Gram-negative bacteria total	30 (61)	Gram-positive bacteria total	19 (39)
		Total number of bacteria	49 (100)

a Percentages may not add up to 100% due to rounding.

**Table 4:** Gender comparison of bacteria and antibiotic resistance.

	Female (n = 4)	Male (n = 12)	p-value
**All bacteria**	2.75±0.95 (0.54–3.57)	2.57±1.61 (1.14–2.74)	0.925
**Gram-positive bacteria**	1.75±0.5 (0.28–1.86)	0.92±1.38 (0.98–2.34)	0.265
**Gram-negative bacteria**	1±0.82 (0.46–3.04)	1.75±0.62 (0.44–1.05)	0.072
**Antibiotic resistant bacteria**	1.19±1.91 (1.08–7.11)	0.74±1.08 (0.76–1.83)	0.563

All results presented as mean number per shark±SD (95% CI).

**Figure 2:**
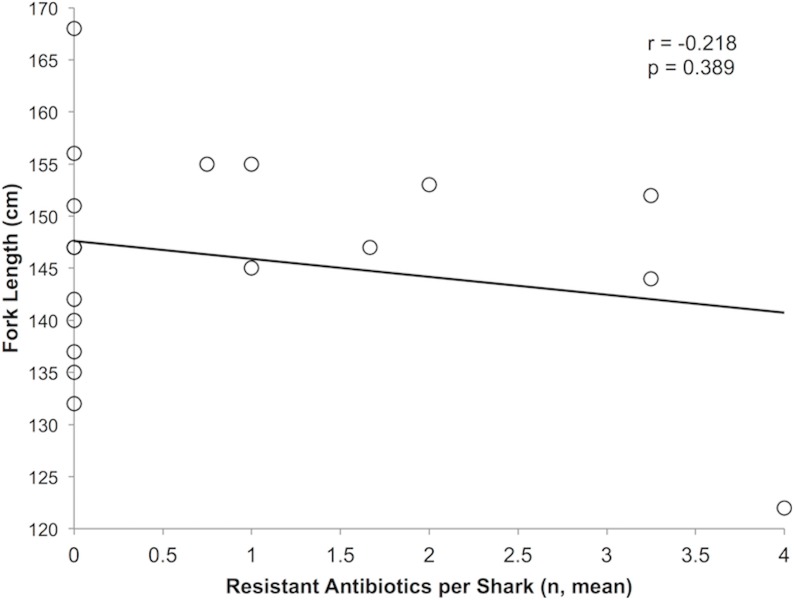
Correlation between the fork length (cm) with the mean number of resistant antibiotics per shark.

Antibiotic susceptibilities for isolated and tested gram-negative and gram-positive bacteria are listed in Tables 5 and 6, respectively. All gram-negative bacteria tested were 100% susceptible to cefotaxime, ceftriaxone, ceftazidime, carbapenems, fluoroquinolones, aminoglycosides, piperacillin/tazobactam and sulfamethoxazole/trimethoprim. All gram-positive bacteria tested were 100% susceptible to fluoroquinolones, daptomycin, linezolid, sulfamethoxazole/trimethoprim (except *Enterococcus faecium*), vancomycin and tetracycline. Oxacillin resistance was observed in 42% and 0% in coagulase-negative *Staphylococcus spp.* and *S. aureus*, respectively. No association was found between fork length and resistance (r = −0.0218, p = 0.389) (Figure 2).

**Table 5:** Antibiotic susceptibilities (%) for gram-negative bacteria isolated in the oral cavity of blacktip sharks^a^.

Bacteria (No. of isolates)	AMP	A/S	AZT	CFZ	CFX	CTZ	CFT	CFM	CIP	IMI	P/T	S/T	TIGE	TOB
**E. coli (1)**	100	100	100	100	100	100	100	100	100	100	100	100	100	100
**Enterobacter spp. (3)**	0	0	100	0	0	100	100	0	100	100	100	100	100	100
**Klebsiella sp. (1)**	0	100	100	100	100	100	100	100	100	100	100	100	100	100
**Moraxella sp. (1)**	100	100	100	100	100	100	100	100	100	100	100	100	-	100
**Pseudomonas sp. (1)**	-	-	0	-	-	100	100	-	100	100	100	100	-	100
**Shewanella** **putrefaciens (1)**	-	-	0	-	-	100	100	-	100	100	100	100	-	100
**Vibrio spp. (14)**	0^b^	100^b^	100	50^b^	50^b^	100	100	50^b^	100	100	100	100^c^	100^b^	100
**Overall Susceptibility** **(No. of isolates susceptible/** **No. of isolates tested)**	25 (2/8)	63 (5/8)	91 (20/22)	50 (4/8)	50 (4/8)	100 (22/22)	100 (22/22)	50 (4/8)	100 (22/22)	100 (22/22)	100 (22/22)	100 (12/12)	100 (7/7)	100 (22/22)

a Pasteurella spp not included in table due to exclusion of susceptibility testing.

b Reported susceptibility is for 2 of the 14 isolates.

c Reported susceptibility is for 4 of the 14 isolates.

**Table 6:** Antibiotic susceptibilities (%) for gram-positive bacteria isolated in the oral cavity of blacktip sharks^a^.

Bacteria (No. of isolates)	AMP	A/S	CFT	CIP	CLIN	DAP	ERY	LZD	OXA	PCN	RIF	S/T	TET	VAN
**CoNS (8)** b	38	38	38	100	38	100	50	100	38	38	100	100	100	100
***E. faecium*** ** (1)**	100	-	-	100	-	100	0	100	-	100	100	-	100	100
***S. aureus*** ** (4)**	25	100	100	100	100	100	75	100	100	25	100	100	100	100
***S. bovis*** ** (1)**	100	100	100	100	100	100	-	100	-	100	100	100	100	100
**Overall Susceptibility (No. of isolates susceptible/No. of isolates tested)**	43 (6/14)	62 (8/13)	62 (8/13)	100 (14/14)	62 (8/13)	100 (14/14)	54 (7/13)	100 (14/14)	58 (7/12)	43 (6/14)	100 (14/14)	100 (13/13)	100 (14/14)	100 (14/14)

CoNS: Coagulase-negative Staphylococcus sp.

a *Micrococcus sp*., *Bacillus sp*., and alpha-hemolytic *Streptococcus sp*. not included due to exclusion of susceptibility testing.

b Includes non-speciated coagulase-negative *Staphylococcus sp*., *S. cohnii-urea*, *S. epidermidis*, *S. hominis*, and *S. scuiri.*

**Figure 3:**
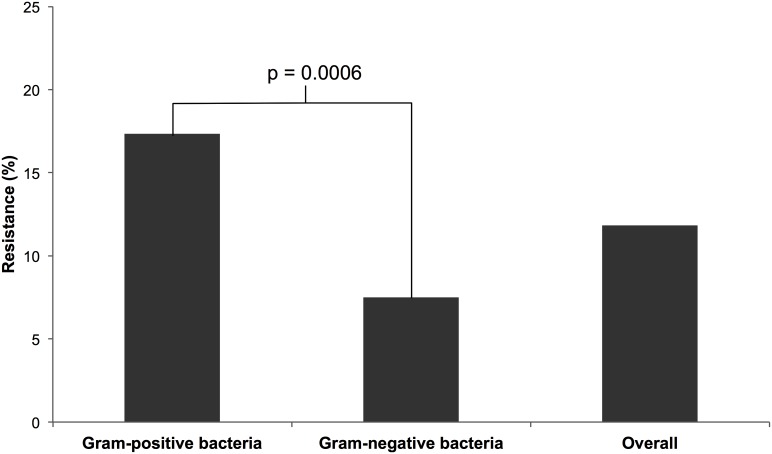
Level of antibiotic resistance in the oral flora of blacktip sharks.

The overall antibiotic resistance rate was 12% for all antibiotics tested (Figure 3). A significantly higher proportion of gram-positive bacteria (17%, 95% CI 77–87%) demonstrated antibiotic resistance than gram-negative (8%, 95% CI 89–95%, p = 0.0006) and 43% of bacteria were resistant to at least one antibiotic. One isolate of *S. hominis* and *Vibrio sp*. met the definition of a multi-drug resistant organism, accounting for 4% of all bacteria. No difference was seen between female and male blacktip sharks regarding the number of resistant bacteria. When specifically extracting and evaluating resistance rates for antibiotics tested in both this study and the comparator [15], no difference in antibiotic resistance rates between blacktip sharks (22%) and tiger sharks (22%, p = 0.883) or bull sharks (17%, p = 0.163) was observed (Table 2).

## Discussion

Blacktip sharks are responsible for many, if not most, shark incidents along Florida’s coast [1], [3]. They are often observed from shore by beachgoers and likely account for a number of unreported minor bites. However, the true number of shark-related incidents is unknown largely due to the fact that experts capable of identifying the proper species are rarely consulted when wounds are just superficial [24]. This fact represents the majority of all incidents worldwide, thus species assumptions are often just based on witness or victim accounts. Furthermore, at least for this study, other closely related spinner sharks (*C. brevipinna*) and silky sharks (*C. falciformis*) are similar in appearance, making a proper identification even more challenging for lay people. Outside of external appearance, teeth morphology is similar amongst the three species, leading to nearly identical wound patterns. Such resemblances lead to confusion when trying to pinpoint the true species involved, resulting in a higher or lower true number of bite incidents by blacktip sharks in Florida waters.

The most commonly isolated bacteria in this study, *Vibrio sp. and Pasteurella sp.,* were anticipated based on the halophilic nature and composition of typical oral flora, respectively. Both species are pathogenic and known to cause life-threatening infections in humans [4], [5], [7]–[10]. Other important human pathogens isolated in this study and known to cause infection include *S. aureus*, non-fermenting gram-negative bacilli (e.g., *Pseudomonas sp.*, *Shewanella putrefacians*) and *Enterobacteriaceae* (e.g., *Enterobacter sp., Klebsiella sp., E. coli*). These enteric gram-negative organisms are not only pathogenic, but may also reflect exposure to sewage effluent. Several of the studies that identified antibiotic resistant enteric gram-negative bacteria with marine animals including sharks, also noted a close proximity to sewage discharge [12], [13], [15], [17], [18], [20]. Nonetheless, when compared to tiger and bull sharks in Brazil [15], the oral samples in our study elicited significantly less bacteria per shark. Additionally, the oral bacterial composition of adult Florida blacktip sharks consisted of significantly less *Enterobacteriaceae* than bull and tiger sharks [15]. It is important to note that sharks sampled in our study were alive, whereas Interaminense et al. [15] transported deceased sharks back to the lab before collecting samples, which may have artificially increased the bacteria density and gram-negative organisms from tissue decay. But even when alive, every mouth swab taken from a shark merely reflects that very moment in a shark’s life. Despite different anatomical swabbing sites, the oral bacterial composition of gram-positive and negative bacteria in this study was similar to the composition of the shark cloaca-anus samples as identified by Blackburn and colleagues [12], although it is unclear if samples from the cloaca or anus can be compared to the oral cavity as no studies have been conducted to that effect.

The amount of rotted tissue present in the oral cavity, thus the density of bacteria, continuously evolves due to the dietary shift throughout the growth of a shark [25]–[27]. Although no comprehensive study exists examining such a shift among blacktip sharks, the food variety sampled from their stomachs makes this assumption likely [28]. Adult blacktip sharks are known to feed on a variety of fish (e.g., pinfish, *Lagodon rhomboides*, pigfish, *Orthopristis chrysoptera*, spotfin mojarra, *Eucinostomus argenteus*, or silver perch, *Bairdiella chrysoura*), which are too large for smaller blacktip juveniles and sub-adults; in addition, younger blacktip sharks inhabit different areas during their earlier stages of life [28]–[31]. Diet composition also needs to be examined from the viewpoint of the preys’ preference, as being herbivores or carnivores, since larger prey can accumulate toxins within their systems. The barracuda, *Sphyraena barracuda*, gathers toxic dinoflagellates (e.g., *Gambierdiscus toxicus*) in its system, especially in its muscles, that causes ciguatera in humans [32], [33]. Should sharks feed on these type of predators their then toxic-laden tissue would likely also be transferred into the oral cavity of a shark, remaining between their teeth until the toxin degenerates, is lost due to teeth replacement, or removed by small-sized sharksuckers, *Echeneis naucrates*, that commonly clean between blacktip sharks’ upper teeth [34].

**Figure 4:**
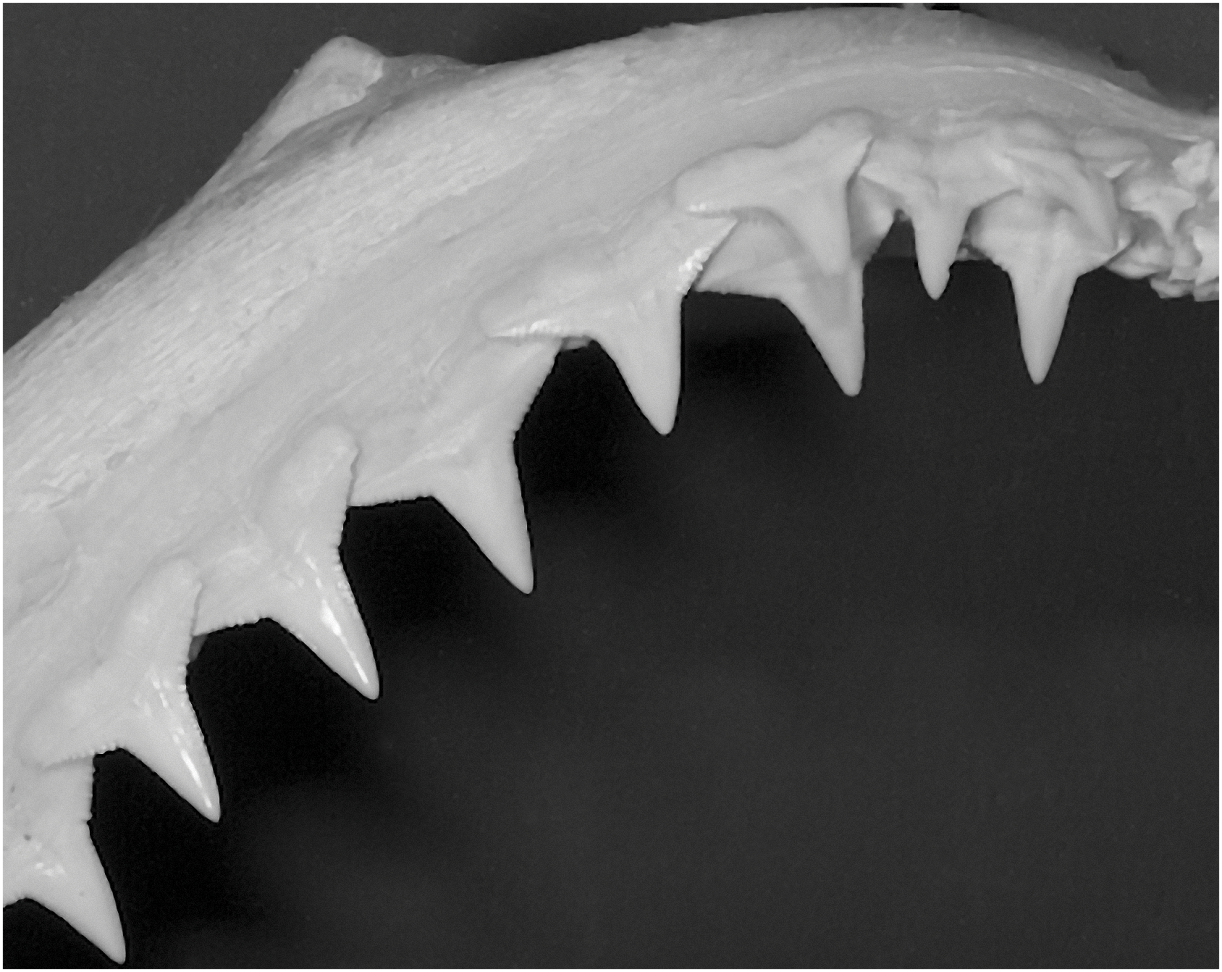
Upper right jaw of blacktip shark.

Compared to blacktip sharks, the larger bull and tiger sharks gouge more often when hunting, inevitably leaving more tissue matter stuck between their teeth as opposed to sucking where prey is swallowed whole. Under such a premise it is obvious that in order to pursue any bacterial count work among shark species, not just a clear understanding about their preferred way of feeding is needed but also their tooth morphology and accompanying overlapping of teeth. In blacktip sharks, the upper anterior and lateral teeth vary in overlap close to the base, which is not observed with lower teeth (Figure 4). This is species specific but very similar in closely related shark species. Although blacktip sharks possess rather slender upper teeth, there is sufficient serration to gouge or cut prey pieces in half, enabling tissue matter to get trapped between their upper teeth. Besides overlapping teeth as a main factor for tissue trapping, resting positions for upper teeth also likely plays a role since teeth are withdrawn (i.e. resting position) into a fleshy groove, which likely festers the production of bacteria, as well.

It is essential to consider the geographic influence on diet since bacterial composition likely also varies accordingly [35]–[38]. The impact of geography on oral bacteria and resistance is still uncertain but one study [12] reported that nurse sharks caught in Belize and the Florida Keys exhibited a similar antibiotic resistance pattern from cloaca-anal swabs, but a seemingly higher bacterial count per sample was seen in the nurse sharks caught in the Florida Keys. A study [39] comparing antibiotic resistance in Atlantic bottlenose dolphins (*Tursiops truncatus*) in Florida and South Carolina found overall resistance was comparable; however, there was a significant difference in the resistance patterns of select antibiotics (e.g., higher rates of resistance to piperacillin, tetracycline and trimethoprim/sulfamethoxazole among *E. coli* isolates in Florida dolphins). Blacktip sharks routinely follow baitfish along Florida’s coast, mainly during early spring months; moreover, this is important because blacktip sharks are highly migratorial, thus their geographical distribution will nearly constantly reflect a different bacterial composition in their oral cavity [40]. The prey variety is dependent upon the degree of geographic distribution of the shark species. This is especially true for cosmopolitan species like the blacktip shark represents. But even if some site fidelity is noticed, food variation will, as mentioned above, be based on the availability of the targeted prey species connected to their respective growth rate and vigilance. The change of prey species composition is not just a question of the shark’s migration pattern or site fidelity but also of the seasonality of available prey.

It remains to be seen how the presented results match with bacteria composition and density sampled throughout the other seasons, or at least over a longer period of time, at alternate geographic locations, as well as through the different ontogeny stages of these sharks. For example, one study [12] suggested that older redfish exhibited a higher rate of resistance than younger redfish. In the current study no association between the shark’s fork length and bacterial resistance was seen; however, based on the fork length range, no juvenile blacktip sharks were sampled thus an association between age and bacteria or antibiotic resistance could not be assessed. On the basis of gender, female and male blacktip sharks did not differ regarding bacterial content or antibiotic resistance, which is in agreement with that reported by Blackburn et al. [12] for bull sharks, yet the sample size was likely too small to detect a difference.

The overall level of antibiotic resistance observed in our study was comparable to similar published studies [12], [15]; however, only 4% of isolates met the definition of a multi-drug resistant organism. Compared to the Brazilian study [15], ciprofloxacin resistance rates were significantly higher in tiger (18%, p = 0.008) and bull sharks (23%, p = 0.002) when compared to blacktips (0%). For gram-positive organisms, bull sharks had exhibited a significantly higher rate of rifampin resistance (50%, p = 0.002) than in blacktips, whereas tiger sharks had a higher rate of tetracycline resistance (33%, p = 0.018). Blacktip isolates were 100% susceptible to both rifampin and tetracycline [15]. None of the blacktip sharks harbored resistance to carbapenems, aminoglycosides, or ceftazidime as one seen in other studies [12], [15]. This is important as the aforementioned antibiotics are often employed for infections caused by multi-drug resistant organisms, including hospital-acquired infections.

The intent of empiric antimicrobial therapy is to provide the most appropriate antibiotics to cover the pathogens most likely encountered for a given infection; moreover, based on our results, empiric therapy for adult blacktip shark bites should be designed to target *Vibrio sp*., *Pasteurella sp*., *S. aureus* and enteric gram-negatives. Although isolated, but due to low pathogenicity in skin infections, coverage against *Bacillus sp., Micrococcus sp.*, and coagulase-negative *Staphylococcus sp*. may not be necessary and would likely be adequately addressed by irrigation during surgery or general medical treatment. Antimicrobials determined to be therapeutic options as single agents for the highest percentage of isolated bacteria are ciprofloxacin, levofloxacin and a 3^rd^ generation cephalosporin such as ceftazidime or ceftriaxone. Additional empiric regimens that may be considered include combinations of a 3^rd^ generation cephalosporin with a fluoroquinolone or tetracycline. Likely also important to consider is the skin flora of the patient when selecting empiric therapy, which may contaminate the wound, or other potentially non-sterile materials (e.g., beach towels, shirts) used during the initial care by lay person prior to medical care. Dependent upon local community incidence of methicillin-resistant *S. aureus* (MRSA), the addition of an antibacterial with MRSA activity may be appropriate.

This study about the identification of bacteria in the oral cavity of a blacktip shark has to be seen as a first attempt, lacking a thorough understanding about the composition of bacteria throughout the shark’s life. There are several limitations to the study that must be acknowledged. First, sampled sharks were seasonally and geographically limited, thus application of the results to other areas or seasons is uncertain. Second, based on the size of the captured sharks in this study, only adult blacktip sharks were sampled. It is unknown if juvenile blacktip sharks would demonstrate similar antibiotic resistance patterns or bacteria composition. Although analysis suggested no difference between female and males, a larger sample size is desired to confirm these findings. Also, while every effort was made to swab all essential anatomy within the oral cavity, it is possible that certain bacteria were not captured during the culturing process or were not viable for adequate growth. To best understand the impact of blacktip shark ontogeny and prey composition on oral bacteria and antibiotic resistance, analysis of the stomach contents for swabbed sharks is needed in future studies. Such sampling will reveal most if not all the bacteria species a blacktip shark likely carries in its oral cavity during any time of its ontogeny. However since the above-mentioned closely related species (i.e. spinner and silky sharks) are not easy to differentiate from blacktip sharks, sampling of those two species also seems prudent to get a thorough understanding of involved bacteria.

## Conclusion

Antibiotic resistant bacteria are present within the oral flora of adult blacktip sharks in Florida; however, the bacteria composition and antibiotic resistance fluctuate and these results only reflect a moment in the shark’s life. Several factors may influence the oral flora throughout its ontogeny, including age, diet, gender and geographic location, all of which require further investigation. Given the worldwide concerns of antimicrobial resistance, research should continue to monitor this trend in marine animals as well. In the instance a person falls victim to an adult blacktip shark, empiric treatment with either a fluoroquinolone or combination of a 3^rd^ generation cephalosporin plus doxycycline is recommended.
